# Musical Role Asymmetries in Piano Duet Performance Influence Alpha-Band Neural Oscillation and Behavioral Synchronization

**DOI:** 10.3389/fnins.2019.01088

**Published:** 2019-10-15

**Authors:** Auriel Washburn, Irán Román, Madeline Huberth, Nick Gang, Tysen Dauer, Wisam Reid, Chryssie Nanou, Matthew Wright, Takako Fujioka

**Affiliations:** ^1^Center for Computer Research in Music and Acoustics, Department of Music, Stanford University, Stanford, CA, United States; ^2^Department of Computer Science and Engineering, University of California, San Diego, San Diego, CA, United States; ^3^Wu Tsai Neurosciences Institute, Stanford University, Stanford, CA, United States

**Keywords:** EEG, neural oscillation, alpha oscillations, perceptual-motor coordination, role asymmetries, social neuroscience, interpersonal coordination, musical performance

## Abstract

Recent work in interpersonal coordination has revealed that neural oscillations, occurring spontaneously in the human brain, are modulated during the sensory, motor, and cognitive processes involved in interpersonal interactions. In particular, alpha-band (8–12 Hz) activity, linked to attention in general, is related to coordination dynamics and empathy traits. Researchers have also identified an association between each individual’s attentiveness to their co-actor and the relative similarity in the co-actors’ roles, influencing their behavioral synchronization patterns. We employed music ensemble performance to evaluate patterns of behavioral and neural activity when roles between co-performers are systematically varied with complete counterbalancing. Specifically, we designed a piano duet task, with three types of co-actor dissimilarity, or asymmetry: (1) musical role (starting vs. joining), (2) musical task similarity (similar vs. dissimilar melodic parts), and (3) performer animacy (human-to-human vs. human-to-non-adaptive computer). We examined how the experience of these asymmetries in four initial musical phrases, alternatingly played by the co-performers, influenced the pianists’ performance of a subsequent unison phrase. Electroencephalography was recorded simultaneously from both performers while playing keyboards. We evaluated note-onset timing and alpha modulation around the unison phrase. We also investigated whether each individual’s self-reported empathy was related to behavioral and neural activity. Our findings revealed closer behavioral synchronization when pianists played with a human vs. computer partner, likely because the computer was non-adaptive. When performers played with a human partner, or a joining performer played with a computer partner, having a similar vs. dissimilar musical part did not have a significant effect on their alpha modulation immediately prior to unison. However, when starting performers played with a computer partner with a dissimilar vs. similar part there was significantly greater alpha synchronization. In other words, starting players attended less to the computer partner playing a similar accompaniment, operating in a solo-like mode. Moreover, this alpha difference based on melodic similarity was related to a difference in note-onset adaptivity, which was in turn correlated with performer trait empathy. Collectively our results extend previous findings by showing that musical ensemble performance gives rise to a socialized context whose lasting effects encompass attentiveness, perceptual-motor coordination, and empathy.

## Introduction

As humans, we face situations every day that demand coordination of our actions with those of other individuals, often in order to achieve a shared goal. Fast-paced and dynamically adaptive sensorimotor interaction can be seen for example, in someone rushing through a crowded airport to catch a flight or a team of paramedics working together to respond to a medical emergency. Here, individuals have to constantly perceive the ongoing actions of others in order to efficiently organize and perform their own actions.

Research investigating the behavioral dynamics that occur between an individual’s actions and the environmental events they perceive has provided valuable insight into how behavioral coordination is achieved (Schmidt and O’Brien, 1997; Richardson et al., 2005; Schmidt et al., 2007). Specifically, research on perceptual-motor coordination has demonstrated that individuals often naturally synchronize and coordinate their limb and body movements with periodic environmental events via visual (e.g., Giese et al., 1996), haptic (e.g., Jeka et al., 1998), or auditory (e.g., Repp and Penel, 2004; Repp, 2006) information. A large number of studies have demonstrated that actor-environment coordination is governed by dynamical processes of entrainment, which generally involve close temporal synchronization to an external rhythm (e.g., Kelso et al., 1990; Wimmers et al., 1992; Schmidt and Turvey, 1994; Byblow et al., 1995; Russell and Sternad, 2001; Wilson et al., 2005). Coordinated human joint action contains many of the same characteristics observed within actor-environment coordination. However, the bi-directional coupling inherent to interpersonal coordination commonly results in a mutual influence between interacting individuals. As a result, the patterns of interaction exhibited during interpersonal perceptual-motor coordination are often dynamic.

As noted by Keller (2008), ensemble music performance highlights the ability of humans to achieve temporally precise interpersonal coordination while also being flexible. Keller proposes that three fundamental skills support this kind of interactive behavior: anticipation, the perception of self and other behavior in relation to the joint goal, and adaptation. The relative symmetries and asymmetries between co-actors appear to be one of the primary factors that influence these processes and ultimately shape musical interaction as well as interpersonal interaction in general.

Relative asymmetries between co-acting individuals can arise from a unidirectional informational coupling between co-actors such that one actor receives information about the other’s behavior but not vice versa (e.g., Goebl and Palmer, 2009; Washburn et al., 2015). There can also be explicit asymmetries in the intrinsic behavioral component dynamics (i.e., resonant limb/movement frequencies, see Washburn et al., 2014). For instance, pianists who exhibit similar preferred tempi during solo performances achieve better temporal synchronization and exhibit greater adaptation to each other during duet performance than pianists who have more divergent preferred solo performance rates (Loehr and Palmer, 2011; Zamm et al., 2015). While these sources of informational and physical asymmetry clearly play a role in shaping joint action, “functional asymmetries,” contextually relevant differences in co-actor roles that can emerge with or without explicit instruction or intention (Richardson et al., 2016), are likely the most common type of asymmetry in everyday interpersonal interaction.

For example, Demos et al. (2017) found that introducing a confederate duet partner to participants as an experimenter vs. a fellow participant introduced an asymmetry in social status. Although this had a minimal effect on temporal coordination during the duet task, participants perceived their synchronization with the “experimenter” as much more successful. The researchers suggest that this effect may have occurred because participants believed that the experimenter’s part was especially important and therefore paid more attention to the confederate’s performance than participants who thought the confederate was another participant.

People also typically conceive that the members of a string quartet have distinct roles corresponding to their part/instrument that are generally related in a hierarchical fashion (i.e., the first violinist is the leader). Such explicitly prescribed functional asymmetries may indeed guide behavior of the whole group. By experimentally manipulating the leader-follower roles within string quartets such that each instrumentalist had the opportunity to act as the leader of the ensemble, Chang et al. (2017) were able to examine the magnitude and direction of information flow between assigned leaders and assigned followers. Their findings indicated that for a given quartet performance the influence of leader behavior on follower behavior was greater than the influence of followers on the leader, as well as the influence of followers on each other.

However, roles and relationships between group members are often not as static as instrument-specific roles would suggest, depending on how music is written, interpreted, and performed at a moment-to-moment basis. For instance, sometimes someone other than the first violinist will provide cues to the members of the group, acting as a “leader.” At other times, the first violinist might engage in repeated turn-taking with another group member, resulting in periods of relative musical role symmetry between the two performers in that their contributions are balanced and equal. Thus, for ongoing interactions that involve numerous opportunities for information exchange between asymmetries seem to vary dynamically and are likely to be shaped by multiple factors. Wing et al. (2014) demonstrated this by examining the emergence of functional asymmetries between quartet members in two separate professional string quartets. They found that when performing the same piece of music, the members of the two separate quartets exhibited unique patterns of symmetry and asymmetry at the beat-to-beat timescale. It therefore appears that instrument-specific-role is not the only functional asymmetry determining temporal coordination on the beat-to-beat timescale. What remains unclear are the ways in which different relational factors in music and performers (e.g., instrument, rhythmic similarity, melodic similarity, performer personality traits) might influence the temporal asymmetry in co-performer activity, as well as how these factors interact to shape this relationship.

Within the past decade researchers have conducted a large number of empirical studies aimed at establishing how neural activity supports the emergence and maintenance of coordinative patterns in joint action. The use of electroencephalography (EEG), and its magnetic-counterpart magnetoencephalography (MEG), allow neuroscientists to observe neural activity with high temporal resolution. In particular, the modulation of spontaneously occurring neural oscillations is thought to constitute one of the principal mechanisms for the dynamic coordination of functions across the brain. Fronto-central alpha rhythms (8–12 Hz) along with central beta-band oscillations (∼20 Hz) are sometimes referred to as “mu rhythms” and appear to play a large role in sensorimotor activities. Specifically, their suppression, or event-related desynchronization (ERD), is observed during voluntary movement (Pfurtscheller, 1992; Salmelin et al., 1995; Babiloni et al., 1999; Taniguchi et al., 2000; Jurkiewicz et al., 2006). Pfurtscheller et al. (1997) related this desynchronization to movement initiation, noting that it began prior to movement onset and was followed by event-related synchronization (ERS), or a return to baseline activity, 2 s following a movement onset.

Interestingly, ERD in central alpha rhythms is also exhibited during imagined movements (Pfurtscheller et al., 2006). In fact, alpha ERD was first detected in individuals watching films of biological motion (Gastaut and Bert, 1954). This effect has been replicated in several subsequent studies (Cochin et al., 1998, 2001; Hari et al., 1998; Martineau and Cochin, 2003; Holz et al., 2008; Arnstein et al., 2011). In their original study, Gastaut and Bert (1954) also noticed that the magnitude of alpha desynchronization increased in relation to how much an individual identified with the actor in a film. Additional work has shown that alpha desynchronization during action observation is modulated by the observer’s action experience. For example, individuals who are given the opportunity to interact with a novel tool or object show greater alpha desynchronization than participants who don’t have the same direct experience when they observe someone else engage with the tool or object (Cannon et al., 2014; Quandt and Marshall, 2014). Professional athletes also display patterns of alpha desynchronization that are distinct from non-athletes when observing videos of their area of expertise (Orgs et al., 2008; Babiloni et al., 2009, 2010).

Given the notable associations between alpha rhythm desynchronization and both voluntary movement and action observation, it is not surprising that these oscillations are also responsive to social, interactive behaviors. Recent dual-EEG studies examining the oscillatory neural activity of co-acting individuals have shown both within- and between-brain coherence in frontal and central alpha rhythms during cooperative, coordinative interaction (e.g., Cui et al., 2012; Sanger et al., 2013). This kind of dual-EEG recording, or “hyperscanning,” has also allowed researchers to identify many different factors associated with alpha modulation in each of the co-actors involved in an interaction. Tognoli et al. (2007), for example, observed desynchronization of right centro-parietal alpha rhythms when participants engaged in a simple finger-tapping task in a social context, suggesting that this decrease in oscillatory power may support somatosensory awareness of a perceived co-actor.

Findings connecting alpha desynchronization to action, action-observation and interactive behaviors have also linked alpha desynchronization to the theoretical human mirror-neuron system (MNS) (Iacoboni and Dapretto, 2006; Oberman et al., 2007; Perry and Bentin, 2009; Frenkel-Toledo et al., 2014; Hobson and Bishop, 2016). A number of studies that relate alpha modulation to MNS activity have revealed desynchronization in right centro-parietal areas during social interaction (e.g., Tognoli et al., 2007; Dumas et al., 2010, 2012; Naeem et al., 2012a, b). However, alpha ERD has also been observed in a variety of other regions during interactive behavior including left centro-parietal, frontal, central, and central midline areas (Lachat et al., 2012; Ménoret et al., 2014; Konvalinka et al., 2014; Ahn et al., 2018). This emphasizes the importance of alpha’s role in supporting social interactive behaviors through domain-general regulatory functions rather than domain- and location-specific sensorimotor processes. Of particular interest here regarding the functional significance of alpha is its apparent role in regulating the dynamic desynchronization and selection of cortical states (e.g., Jin et al., 2006; Klimesch et al., 2007; Klimesch, 2012). This links alpha closely to mental states of alertness, expectancy and attention (e.g., Klimesch et al., 1998; Pfurtscheller, 2003; Perry and Bentin, 2009), and to the temporal coordination of intrapersonal and interpersonal behavior often supported by these states in the context of interpersonal interaction. This is consistent with Novembre et al. (2016) observation that alpha desynchronization occurred during periods of strong temporal entrainment between co-actors during piano duet performance. Existing findings therefore indicate that alpha ERD is likely to occur frequently in both individuals involved in a joint action task.

Interestingly, the evolution of alpha desynchronization during temporal coordination is related to asymmetries in co-actor roles (Konvalinka et al., 2014). Specifically, for pairs engaged in a synchronized finger-tapping task, the individual who exhibited less adaptive, or more leader-like, behavior generally displayed greater alpha desynchronization in frontal brain regions. This demonstrates that the dynamics of alpha desynchronization are sensitive to subtle, emergent asymmetries between interacting individuals. Relatedly, Sanger et al. (2013) observed that the coherence between frontal alpha oscillations in co-performing guitarists was stronger for leader-to-follower coupling than for follower-to-leader coupling. In Sanger et al. (2013) study the roles of leader and follower were explicitly assigned to co-performers prior to duet performance.

Hari and Kujala (2009), among others, have pointed out the apparent links between alpha ERD and the neural processes that support action observation and ultimately interpersonal interaction, including motor imitation, emotional contagion, and empathy. Babiloni et al. (2012) were the first to empirically investigate the relationship between alpha ERD and empathy by having saxophonists observe their own previously recorded ensemble performances. The findings from this study revealed that the musician with the highest score on the self-report empathy measure showed widespread alpha desynchronization during performance observation. Perspective-taking also appears to be related to perceptual accuracy in the context of action-observation. Work by Engel and Keller (2011), for example, has shown that for individuals viewing point light displays (PLDs) of improvised or imitated musical performances, the person’s accuracy for identifying the type of performance was positively correlated with scores on a self-report measure of perspective-taking. Relatedly, Pecenka and Keller (2011) observed that individuals who reported higher levels of perspective-taking behavior showed greater anticipation of tempo-changing metronome sequences. Collectively, existing work on empathy and social interaction reveals strong links between trait empathy and both (1) alpha desynchronization, and (2) behavioral coordination characteristics.

**FIGURE 1:**
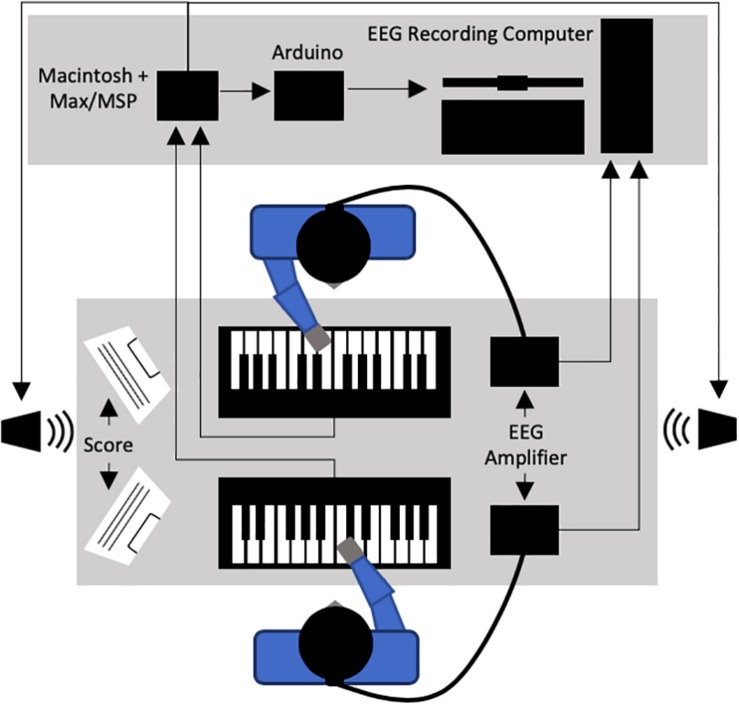
General experimental set-up for the current study.

In the current study, we used the context of piano duet performance to evaluate patterns of behavioral and neural activity under conditions of asymmetry between co-performers. To do this, we experimentally introduced three different task-specific asymmetries: (1) musical role (starting vs. joining), (2) task similarity (similar vs. dissimilar melodic parts), and (3) performer animacy (human-to-human vs. human-to-non-adaptive computer). The musical tasks used in the current study were piano duets, played by two players face-to-face, each with an electronic keyboard (see Figure 1). The piano duet scores were composed by our research team, consisting of simple short melodies designed so that each of the three experimentally introduced asymmetries were experienced during the initial portion each trial prior to the final unison period. In this final unison period of four notes, co-performers always had identical musical tasks.

We designed this experimental task to support a multi-faceted study which allowed our team to perform two separate investigations. In the investigation presented here, we evaluated neural alpha and behavioral activity during the final unison measure of the duets to determine how the asymmetries in the preceding portion of the task served as a priming context and shaped the interaction of subsequent unison performance. The other investigation, described in Huberth et al. (2019), was focused on performers’ neural responses to outcome expectation violations when altered pitch feedback was experimentally introduced within the first four measures of the turn-taking duet performances. Specifically, the authors compared the feedback-related negativity (FRN) and P3 complex within each individual’s EEG data in response to altered pitches in one’s own part vs. the co-performer’s part. Generally, performers exhibited greater responses to alterations corresponding to their own part, especially when their part was melodically similar to their co-performer’s. Our current investigation is based on behavioral and EEG data from only the fifth and final measure of the duets where no altered pitch feedback was used and the notes performers had to play stayed identical across the different conditions experienced in the preceding part of the duet. Thus, the task was designed to accommodate two investigations with one round of data collection with each investigation ultimately involving unique data analysis.

Based on the above literature, we had several hypotheses about the reactivity of alpha activity and performers’ coordinative behaviors in our task. First, however, it is important to note that differences in starter and joiner movement onset immediately before the unison prevented us from directly comparing the effects of performing the starting vs. joining role on note-onset synchronization or alpha desynchronization. Because starting performers played three notes immediately prior to the unison part, they would show more pronounced alpha desynchronization during the unison. Second, we hypothesized that performer animacy would have a significant effect on the stability of note-onset synchronization. This expectation was informed by Fairhurst et al.’s (2013) findings that the stability of human tapping with a virtual partner was influenced by the adaptability of the partner, with both low and high adaptability leading to low synchronization stability. Based on work by Billeke et al. (2014) we also expected that individuals might show greater neural responsiveness in the alpha oscillations during interaction with a human partner compared to a computer partner.

As for the task similarity, we are not aware of existing studies illustrating the effects of any type of task similarity on neural activity during joint action. On the one hand, the finding that action familiarity has an effect on neural measures during action imagery and observation as well as interaction (Orgs et al., 2008; Babiloni et al., 2009, 2010; Cannon et al., 2014; Quandt and Marshall, 2014; Novembre et al., 2016) indicates that greater melodic similarity between parts may engage more neural resources in duet performing musicians. On the other hand, Skewes et al. (2015) observed that task asymmetries for a dyadic aim and click task had minimal effects on co-actor synchronization, except in cases where the level of difficulty was substantially different between co-actors. Thus, we expected that the effect of having similar or dissimilar musical tasks might be the most subtle of the three asymmetries we manipulated in the current study. However, as shown in Babiloni et al. (2012), we also saw this manipulation as highly relevant to trait empathy and an important factor for understanding asymmetries in musical interaction and social interaction in general and were interested to see how it might shape coordinative activity in interaction with the other two asymmetries we examined.

Lastly, we would like to note that our investigation on the effects of task-specific asymmetries on temporal coordination behaviors during an experimentally-controlled musical duet task has implications for both musical and non-musical everyday interactions. Our study design allowed us to unambiguously examine how some of the asymmetries that are common to ensemble music performance might interact to shape musical interaction, which contains various relationships between performers that emerge and change dynamically as music unfolds. Notably, our study also has implications for interaction dynamics beyond musical performance. Any time multiple individuals coordinate to achieve a shared goal there will be some discrepancies between individuals in their attentiveness and adaptivity to one another as well as in their behavioral timing. Asymmetries between individuals can lead to these kinds of discrepancies, and the evolution of discrepancies over time will shape the course of interaction. Returning to our example of the paramedic team, one can imagine how the collective activity of the team will be influenced by one member arriving before the others, the need for team members to address an individual’s multiple, similar or different injuries, and the need for each team member to multi-task, limiting their ability to attend and adapt quickly to team member actions. These interpersonal asymmetries and others will have a significant impact on how each member of the team experiences the actions of their team members and acts to support team success. By gaining a greater understanding of the effects of co-actor asymmetries we therefore broaden our understanding of how individuals interact to achieve collective goals.

## Materials and Methods

### Participants

Twenty-four pianists (twelve pairs) were recruited from the Stanford University community for participation in this study. Nine pianists were removed from data analysis due to technical failures (*N* = 3), high behavioral error rates (*N* = 4), and excessive data artifacts (*N* = 2). Compared to the participant data analyzed in Huberth et al. (2019), we removed three additional pianists because of errors (*N* = 2) and artifacts (*N* = 1) that specifically affected our analyses. The four pianists with the high error rates comprised two pairs. Results reported here are therefore for a sample of 15 pianists (*M* = 14.33; *SD* = 4.92 years musical experience). These pianists ranged from 18 to 28 years of age.

Of the pairs recruited, two pairs knew each other and had played duets together prior to the experiment. Only one of these pairs’ data was included following the removal of participant data prior to analysis as described above. The remaining pianists met for the first time during participation in the study. All pianists were right-handed except for one, who was not included in the data for analysis. The study protocol was approved by the Stanford University Institutional Review Board and participants provided written informed consent. Pianists were paid $20/h for their participation.

### Apparatus

Two Yamaha Axiom-61 digital keyboards were arranged facing each other on a table within a sound-shielded room in the lab (see Figure 1). Two loudspeakers were used to provide auditory feedback to the performers during the study, with one placed at each end of the table. A custom module for Max/MSP 7.0.1 run on a Macintosh computer (OSX 10.9.5) was used to control all auditory feedback throughout the study. The piano timbre used throughout and the drum timbre used for introductory metronome clicks were built-in sounds from the OSX MIDI sound synthesizer, AU DLS Synth. All auditory feedback was played at a constant volume of approximately 75 dB SPL throughout the study (i.e., pianists were not able to produce changes in dynamics during performance).

The Max/MSP program was also used to generate trigger codes associated with meaningful timepoints and experimental conditions as they occurred in each task trial. This included tracking pianist performance for note accuracy and inter-onset-interval (IOI) in real time based on the current musical score. These codes were sent through an Arduino Uno to the computer used to record EEG data in order to achieve temporal mapping between each EEG recording and the event time course of the musical task being completed.

The component latencies produced by this apparatus were evaluated by comparing the onset latency of (1) a piano key press, (2) the resulting trigger code produced by Max/MSP, and (3) the associated auditory feedback using simultaneous three-channel audio recording of all three events (see Wright et al., 2004). The average key press to trigger code onset was 27 ms (*SD* = 4.0 ms), and the average delay between a trigger code and the associated auditory feedback was 21 ms (*SD* = 3.3 ms).

### Stimulus and Task

**FIGURE 2:**
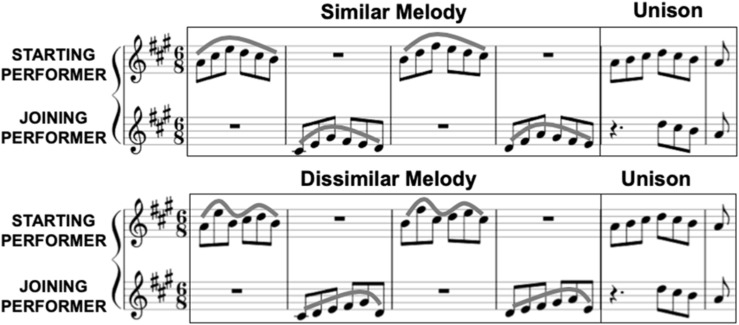
Examples of the similar **(top)** and dissimilar **(bottom)** musical task duets composed for the current study. In each duet the starting and joining performers alternated playing in the first four measures, and played the last four notes of the fifth measure in unison. Melodic contour lines show that in the “similar” task condition the starting and joining performers played similar melodic patterns, while in the “dissimilar” task condition the patterns were distinctly different. The final unison measure was the same across all duets. All of the analyses we conducted in the current study were for neural activity and note-onset behavior during this final measure.

We composed four unique piano duets for the current study. All duets had the same five measure structure (see Figure 2). This included four initial measures in which only one pianist played at a time and the partners alternated each measure. This meant that the “starting” player for a given trial played in measures one and three while the “joining” player played in measures two and four. In two of the four duets the starting and joining parts had similar melodic contours in the first four measures, while in the other two duets the contours were distinctly different (within a given player’s part the two phrases they played alone always had the same contour). Each duet also included a final fifth measure, in which the starting player played the first half alone and both pianists played the remainder together in unison. The notes played in unison were identical for both the starting and joining players and were the same across all four duets. Duets were composed so that each part could be played with the right hand alone and overall hand position could remain the same throughout. Fingering numbers were provided in the score to encourage consistency between participants and ensure minimal movement.

During the study each pianist played the duets with both a human co-performer and a computer partner (audio only). The computer partner was non-adaptive and produced notes with a constant IOI of 500 ms, or a tempo of 120 bpm for the eighth note. Each experimental trial began with three isochronous metronome clicks corresponding to the eighth note, with this same IOI of 500 ms regardless of whether the trial was to be performed by two pianists or one pianist and the computer partner. Following these clicks the starting player began the first measure. After the completion of a trial, the introductory metronome clicks indicating the start of the next trial would begin after a random interval of silence (1.5–2.5 s).

In the first four measures of each duet the auditory feedback associated with key presses was sometimes altered. In other words, even though a pianist had played the correct key based on the musical score a different pitch would be presented as auditory feedback. These manipulations were introduced within both parts of each duet to evaluate the feedback-related negativity (FRN) associated with altered feedback corresponding to a pianist’s own part and their partner’s part as identified using EEG. In each trial, one pitch alteration occurred in each performer’s part. This occurred on either the 4th or 5th note of one of the two phrases each performer played. All alterations produced in-key pitches that were ± two scale notes from the printed score note. Further information about these manipulations and the associated analyses and findings are presented in Huberth et al. (2019).

In the current study, our analysis focused solely on the fifth measure of each duet, which did not contain any altered auditory feedback. It is important to note that we fully counterbalanced the altered note position, pitch direction, and frequency of occurrence of the altered feedback such that these manipulations did not influence the data presented here. The last possible position for altered feedback (in the fourth measure, affecting the joining performer’s part) occurred at least one second before the onset of the data epoch used in the current study. For the present study we established that there was no significant difference in either starter or joiner behavior when there was an alteration in the fourth measure compared to when there was not. Thus, we have collapsed data across these conditions. Using this design we were able to assess the influence of asymmetries in (1) musical role (starting vs. joining), (2) task similarity (similar vs. dissimilar melodic parts) and (3) partner animacy (human vs. computer) experienced in the preceding part of the piano duet performance on behavioral and neural activity during joint performance of the same musical sequence within the fifth measure. All analyses presented in the current paper are for the time period associated with this final unison measure.

### Procedure

One pianist from a pair was randomly selected to arrive at the lab first, be prepared for EEG recording, and complete the first half of the study with the computer partner. While this individual was performing with the computer partner, the other pianist arrived in the lab and was prepared for EEG recording. When the initial pianist was finished playing with the computer partner, both pianists played together and then the second pianist completed the latter portion of the study with the computer partner. At the end of each pianist’s recording session we asked them to complete two questionnaires: the 40-question version of the Cambridge Empathy Scale (Baron-Cohen and Wheelwright, 2004), and a custom 10-question measure designed to ascertain each participant’s prior familiarity with their human partner and their experience of the task difficulty.

Upon arrival in the lab each pianist was introduced to the four duet scores composed for the current study. They were asked to memorize the duets as quickly as possible, but were informed that the printed scores would be placed next to their keyboard throughout the study should they need to reference them. All pianists were also asked to keep a fixed gaze at a comfortable location during trials in order to avoid excessive eye movement artifacts in the EEG recording. Before starting the first block of trials each individual was informed that throughout the study the auditory feedback associated with key presses would sometimes be altered and were asked to continue playing even if they heard an incorrect pitch (see previous section).

Each pianist played four blocks of trials with the computer partner and four blocks of trials with their human partner for a total of eight blocks. Partners played a single duet score within each block, with the starting and joining roles fixed throughout the block. It is important to note that the difference in these roles was communicated to participants only with respect to who played first at the beginning of the trial, and was not characterized as a “leader” vs. “follower” musical relationship. Partners played the same duet score for two back-to-back blocks before switching to another score. This allowed us to counterbalance musical role so that each pianist played both the starting and joining role for each duet they played (e.g., if a person played the starting role for a duet in Block 1 the same person would play the joining role for the same duet in Block 2). This organization allowed all pianists involved in the current study to play each of the eight unique musical parts. We also counterbalanced the presentation of task similarity conditions such that each pianist played one melodically “similar” and one melodically “dissimilar” duet with both the human and computer partner. The order of presentation of the specific duet scores was counterbalanced across all participants as well.

The first two trials of every block were treated as practice trials and contained no pitch alterations. These practice trials proceeded directly into the 48 target trials for the block. As noted above, the location, direction and magnitude of altered pitch feedback within the first four measures of each trial was counterbalanced within each block (see Huberth et al., 2019 for details). We used the Max/MSP program to control sound and evaluate performance in each trial. A trial was counted as incorrect if either partner (1) pressed an incorrect key based on the score for that trial or (2) produced an IOI more than 125 ms shorter or longer than the expected IOI (500 ms). When an error occurred, the auditory feedback was stopped immediately to signal to pianists that they should prepare for the next trial. A short period of silence was maintained before the next trial began.

The length of this period was randomly selected from a uniform distribution of values representing each possible integer value in milliseconds between 1.5 and 2.5 s duration. Each trial classified as incorrect was appended to the end of the current block and had to be performed in order for the block to be complete. A single block took approximately 15 min to complete. Max/MSP recorded all note onset timing information from both keyboards as well as the success/error status of all trials in log files, which were later used for behavioral data analysis.

Electroencephalography data were collected either from a single pianist during interaction with the computer partner, or simultaneously from both pianists. All recording took place in a sound-attenuated and electromagnetically-shielded chamber within the lab. A member of the research team monitored participant compliance with stated instructions via a window from an adjacent room. Participants were encouraged to take brief breaks between blocks when needed. An experimental session for a single participant took between 3.5 and 4 h.

### EEG Recording and Preprocessing

Electroencephalography data were collected using whole-head, 64-channel Neuroscan Quik-Caps (10–20 system), a SymAmpRT amplifier, and Curry 7 acquisition software (Compumedics Neuroscan Inc., El Paso, TX, United States). This included the recording of vertical and horizontal electrooculograms (EOG). Recordings were made at a 500 Hz sampling rate. Electrode impedances were kept below 10 kΩ throughout recording. Scalp electrodes on each cap were referenced to a midline electrode between CPz and Cz for recording. Prior to analysis, the raw recordings from each individual were re-referenced using the common average reference for the cap. The SymAmpRT amplifier allows for simultaneous recording from up to four caps, precluding the need for a temporal synchronization mechanism between the EEG recordings from duet partners. We processed and analyzed EEG data in MATLAB (Mathworks Inc., Natick, MA, United States) using custom scripts which incorporated routines from the Brainstorm toolbox (Tadel et al., 2011).

We removed eye artifacts from the continuous EEG data via Source Space Projection routines provided by the Brainstorm toolbox. First stereotypical eye-artifact events (blinks and movements) were detected using a single, continuous representative raw file for each participant. Then using these events a set of projectors for the participant was constructed and applied to each of the participant’s trials in order to remove blink and movement artifacts. This automatically removed projectors that explained a substantial amount of variance in the participant’s data (in our case more than 15% of the time). In addition, we chose to remove any additional projectors with a pattern of largely lateralized topography in order to avoid any spurious effects on the comparison of right and left electrode groupings.

We created epochs using a time window between −1.0 s before and 4.0 s after the onset of the fifth measure (i.e., total epoch duration was 5 s) from correctly-completed trials. This resulted in 48 epochs per condition per participant. Within each epoch, any channels exhibiting peak-to-peak amplitudes ±150 μV were rejected. We employed this channel rejection threshold in the current study so that we could conduct analyses on as many EEG trials as possible, and avoid artifacts resulting from any large amplitude changes. The evoked response for each condition was calculated by averaging trial epochs for that condition across participants, using a baseline period of 50 ms before the onset of the measure.

### Measures and Analyses

In this study we were interested in evaluating the effects of the three co-performer asymmetries introduced experimentally on behavioral and neural activity during musical unison. In order to assess behavioral coordination we evaluated the *Note-Onset Asynchronies* between two co-performers during musical unison. To examine the neural activity exhibited by each individual before and during unison we investigated the occurrence of *Alpha Desynchronization* using EEG. We also measured each individual’s trait *Empathy* so that we could investigate potential associations between empathy, movement asynchrony, and neural alpha desynchronization in our study’s performance task.

#### Note-Onset Asynchronies

For each experimental trial we calculated a Note-Onset Asynchrony measure between players during the concluding unison segment, which consisted of the final four notes in each duet and was identical for both players and throughout all conditions. This analysis required data for both performers within a duet. All behavioral analyses were therefore conducted on the seven complete pairs within our data set (14 participants). We first determined the note-onset time for each of the four notes in the unison segment as executed by each player (i.e., starter and joiner). We then found the asynchrony between players for each note by subtracting the key press time for one player from the corresponding key press time of the other player. We did this separately from each player’s perspective so that we had two series of asynchronies values for each trial, one for the starting player and the other for the joining player. Specifically, for the starting player series we subtracted joiner timing from starter timing and vice versa for the joining player series. As a result, if the current player played a note before the other player, this would result in a negative asynchrony value for the current player and if the current player played after the partner it would result in a positive asynchrony value.

For each trial we used each player’s four-note asynchrony series to calculate two measures: mean asynchrony and standard deviation of asynchrony. We then averaged these single-trial values across the trials corresponding to each musical role × task similarity × partner animacy experimental condition to gain measures of average asynchrony and asynchrony variability. In order to account for possible deviations in tempo between performers, we ultimately divided each performer’s average asynchrony and asynchrony variability for each experimental condition by their average IOI in that condition. We present these measures as the percent asynchrony exhibited based on IOI.

#### Alpha Modulation

We computed normalized power within the alpha frequency band for each combination of musical role × task similarity × partner animacy conditions in three steps. This analysis included all 15 of the participants retained in the current study, coming from a total of eight different duet pairs.

(1) As noted above, single-measure epochs from each participant for a given condition were averaged to produce the associated evoked response for the condition. We then subtracted this within-participant condition average from each participant’s own trial epochs in order to derive the induced response exhibited by each participant in that condition.
(2) We used these resultant epochs to generate time-frequency representations (TFRs) for each epoch using Morlet wavelet decomposition with 32 logarithmically-spaced bins from 1 to 60 Hz. Brainstorm routines were employed to calculate the *z*-score normalized signal power in each bin for a single epoch as the product of each wavelet coefficient and its complex conjugate. We then averaged TFRs across epochs to generate a characteristic TFR for each experimental condition. During this process we observed that for some average TFRs, normalized power seemed to be spuriously concentrated a specific TF region (i.e., very little relative power was observed for the majority of the spectrum). For these averages we made a close examination of each of the contributing epoch TFRs in order to identify the source of the seemingly artifactual relative power concentrations. Oftentimes these were the result of extreme changes in single channel behavior within a few single trials per condition or single trials in which a number of channels simultaneously exhibited noisy behavior. We removed such channels and trials. This affected an average of 2.4 trials per condition, per participant. A total of 97.78% of the original, correctly performed trials are ultimately included in the data presented here.
(3) Using the corrected TFRs we extracted the alpha-band time course in each condition by averaging normalized power across the four existing frequency bins corresponding to the alpha-band (8.2, 9.3, 10.4, 11.7 Hz). This allowed us to establish each participant’s average alpha activity for a given experimental condition, which we low-pass filtered at 8 Hz to remove transients. The resultant time series was baselined using a period of 80 ms before the onset of the measure. We then calculated the average activity within each of three electrode groupings: frontal-centro-medial (fcm: F1, Fz, F2, FC1, FCz, FC2, C1, Cz, C2), parietal left (pl: C1, C3, TP7, CP5, CP3, P7, P5, P3), and parietal right (pr: C2, C4, CP4, CP6, TP8, P4, P6, P8).

Alpha ERD amplitude at the unison onset was the main feature of interest for statistical analysis in the current study. To establish our exact analysis time window we first generated the average alpha ERD waveform associated with the fifth measure, including each of our three electrode groups of interest across both starting and joining roles. We then identified the latency of the negative ERD peak (i.e., trough) closest to the expected unison onset time (i.e., 1.5 s after the onset of the fifth measure).

We established the time window of interest around the time point associated with the unison-related trough using the method suggested by Keil et al. (2014) for selecting a window without bias. The process for identifying this time window required that we first locate the positive peaks on either side of the trough. For the peak preceding and the peak succeeding the trough, we measured the absolute value of the difference in trough-peak amplitude. We then found the time point on the ERD grand average trajectory associated with an amplitude 50% of the total corresponding trough-peak amplitude. Through this process we obtained a time window for evaluating unison onset-associated activity of 1.12 to 2.36 s after the onset of the fifth measure.

#### Empathy Quotient

We used the 40-question Cambridge Empathy Scale (Baron-Cohen and Wheelwright, 2004) to establish each participant’s trait empathy, or empathy quotient (EQ). A higher EQ is indicative of greater empathy toward others.

## Results

### Task Performance Evaluation

Throughout our study we maintained two criteria for successful trials: (1) correct keypresses based on the score and (2) a consistent tempo, defined as every note IOI being between 375 and 625 ms (±25% from the correct IOI of 500 ms). Trials which included violations of either criterion were classified as error trials (see Procedure section for details). To examine whether performer error rates were associated with increasing in fatigue as the study progressed, we compared the number of errors exhibited in the first vs. second half of each study block. This revealed no differences, establishing that participants did not commit significantly more errors later in the block.

In order to further examine the possible deviation of performance tempo from the expected IOI, we also conducted separate 2 (task similarity: similar, dissimilar) × 2 (partner animacy: human, computer) × 7 pair (seven unique performer pairs) mixed-model analyses of variance (ANOVAs) on the average IOI during the analysis time window for each of the starting and joining roles.

For performers assuming a starting role we found no significant interactions between variables, but we did find a significant main effect of partner, *F*(1,6) = 62.50, *p* < 0.001, *η*_*p*_^2^ = 0.90. As can be seen in Table 1, IOIs were shorter when participants played with a human partner.

**TABLE 1:** Performer IOIs during Unison across Experimental Conditions.

		**IOI (ms)**			
		
		**Starter**		**Joiner**	
			
**Partner**	**Task Similarity**	***M***	***SD***	***M***	***SD***
Human	Similar	467.58	17.23	466.82	15.92
	Dissimilar	471.27	14.83	470.82	14.07
Computer	Similar	500.28	5.74	497.89	3.64
	Dissimilar	487.792	3.65	496.71	2.94

For performers assuming a joining role, we found a similar main effect for the partner condition, *F*(1,6) = 75.53, *p* < 0.001, *η*_*p*_^2^ = 0.92. We also observed a significant main effect of pair for this role, *F*(1,6) = 3.95, *p* = 0.047, *η*_*p*_^2^ = 0.77. Fisher’s LSD *post hoc* comparisons revealed that two pairs generally exhibited shorter IOIs than some of the other pairs during the human partner conditions. Therefore, the subsequent analysis of note onset asynchronies was normalized to each performer’s average IOI value in a given condition.

### Note-Onset Asynchronies

We assessed the effects of task similarity and partner animacy in the current study separately for the starting and joining conditions based on the distinct movement requirements preceding unison.

**FIGURE 3:**
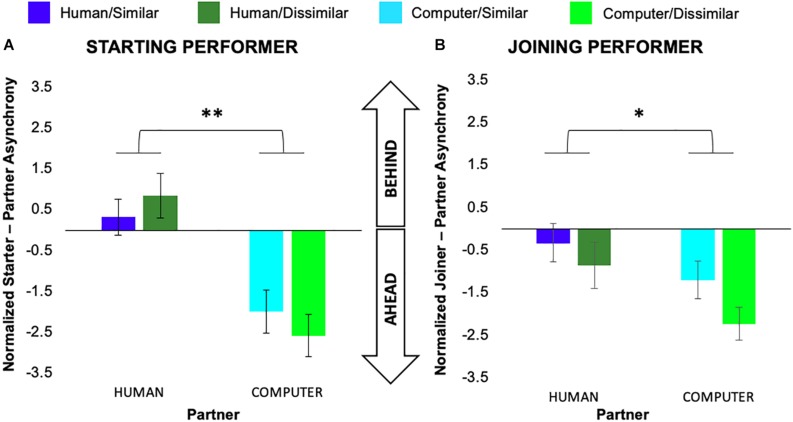
Normalized average asynchronies exhibited by participants in the role of **(A)** starting and **(B)** joining performer. Error bars show standard error. ^∗^*p* < 0.05, ^∗∗^*p* < 0.01.

We conducted separate 2 (task similarity) × 2 (partner animacy) × 7 (pair) mixed-model ANOVAs on the average note-onset asynchrony measure normalized by each performer’s average condition IOI for each of the starting and joining roles. For performers assuming a starting role, there was a significant main effect of partner animacy, *F*(1,6) = 14.49, *p* = 0.007, *η*_*p*_^2^ = 0.67, but no main effect of task similarity or pair or interactions between variables. We observed a similar pattern of results for performers assuming a joining role, again with a significant main effect of partner animacy, *F*(1,6) = 7.70, *p* = 0.03, *η*_*p*_^2^ = 0.52, but no main effect of task similarity or pair or interactions between variables. As can be seen in Figure 3, performer note-onsets generally arrived ahead of the computer partner but were much more closely synchronized to the human partner.

**FIGURE 4:**
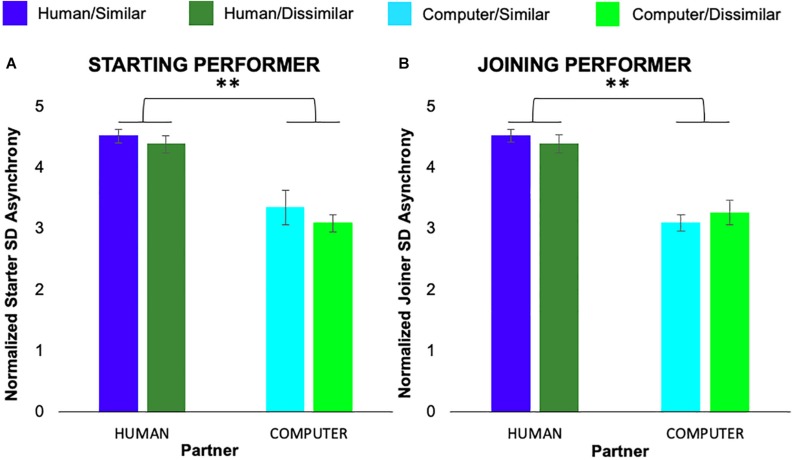
Normalized average standard deviation of asynchronies exhibited by participants in the role of **(A)** starting and **(B)** joining performer. Error bars show standard error. ^∗∗^*p* < 0.01.

We also conducted separate 2 × 2 × 7 ANOVAs on the standard deviation of average note-onset asynchrony measure for starting and joining performers. For starting performers we found a significant main effect of partner animacy, *F*(1,6) = 29.96, *p* = 0.001, *η*_*p*_^2^ = 0.81, but no main effect of task similarity or pair or interactions between variables. For joining performers, we also found a main effect of partner animacy, *F*(1,6) = 57.26, *p* < 0.001, *η*_*p*_^2^ = 0.89, but no main effect of task similarity or pair or interactions between variables. Figure 4 shows both starting and joining performers exhibited greater variability in note-onset asynchronies when playing with a human co-performer as compared to the computer.

### Alpha Desynchronization

**FIGURE 5:**
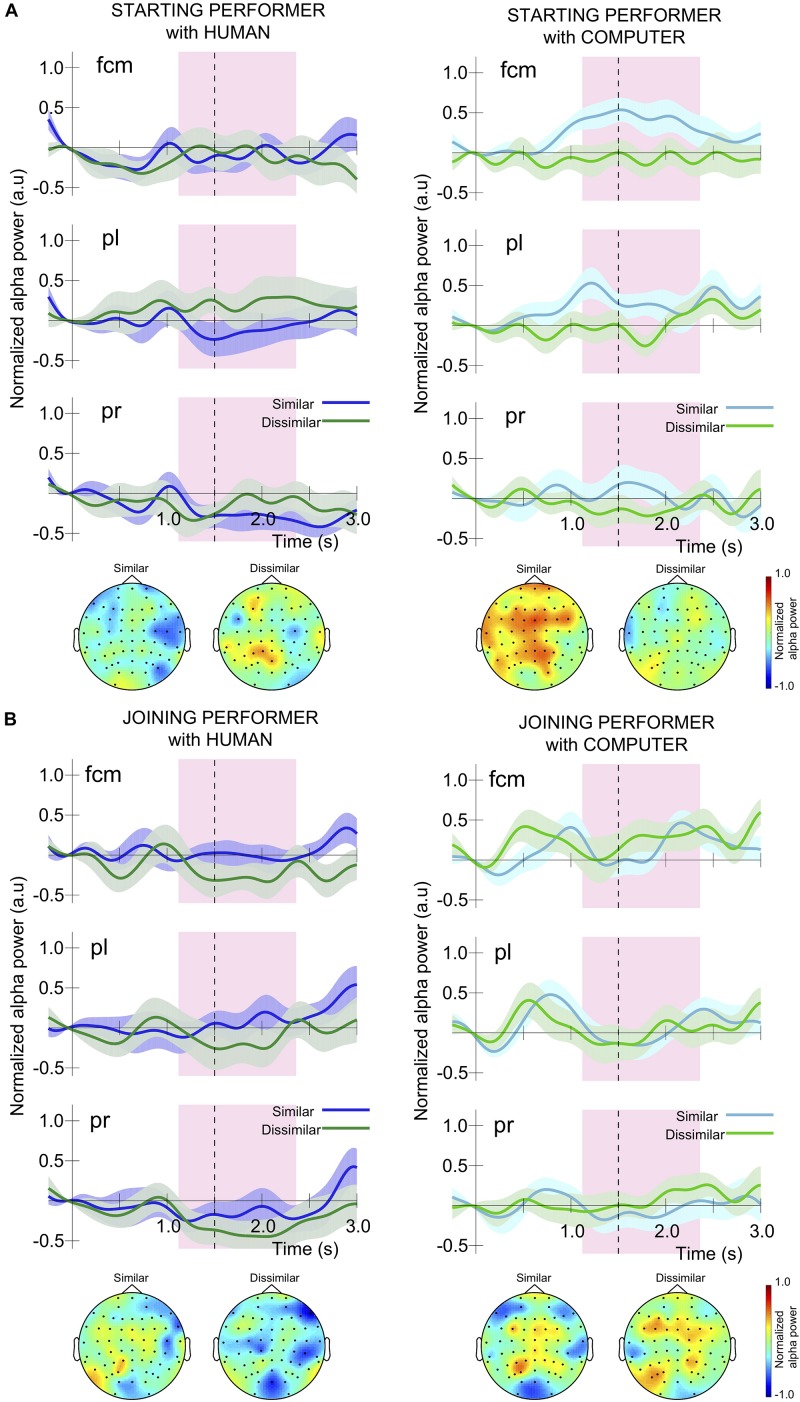
Time course of grand average normalized alpha power around the final unison measure for participants in the role of **(A)** starting and **(B)** joining performer, in the three electrode groups, fcm, pl, and pr. The onset of the measure is designated as time 0 and the starter played three notes before the joiner started playing. The fourth note of the measure, and first note of unison, occurred around 1.5 s and is marked by a black dashed vertical line. The shaded purple rectangle corresponds to the time window around unison used for statistical comparison between conditions (1.12 to 2.36 s). The topography associated with this time window in each condition is also provided. Shading around the alpha power time course for each condition corresponds to the standard error.

We conducted separate 2 (task similarity) × 2 (partner animacy) × 3 (electrode group: fcm, pl, pr) × 8 (pair) mixed-model ANOVAs on alpha activity for each of the starting and joining roles. As we have discussed in the “Measures and Analyses” section, it was necessary for us to use just the seven full pairs for the behavioral analyses, but we included all 15 participants in the alpha analyses resulting in eight-levels for the pair factor. A time window of 1.12 to 2.36 s after the onset of the fifth measure was used to capture activity related to the unison onset which started on the fourth note of the measure, at approximately 1.5 s. Figure 5 illustrates the time course of normalized alpha power during this fifth measure.

As can be seen in Figure 5A, the alpha modulation range for performers in a starting role during the unison measure was often small because they were already moving during this time window. When pianists played with a human partner these fluctuations reflected the temporal regularity of the note-onset actions as well as slightly larger desynchronizations prior to the unison onset in some conditions, but did not reveal substantial differences between having similar or dissimilar musical parts. In contrast, for starting performers playing a similar part to their computer co-performer there was a large alpha synchronization prior to the unison onset, leading to a sustained difference in alpha activity compared to performance of dissimilar musical parts across the time window of interest. A 2 × 2 × 3 × 8 ANOVA conducted on alpha modulation for performers playing the starting part revealed a significant interaction of task similarity and partner animacy, *F*(1,7) = 5.82, *p* = 0.047, *η*_*p*_^2^ = 0.45, but no other interactions or main effects. To explore this interaction we collapsed across the electrode groupings, released pair as a factor and conducted a simple effects analysis evaluating the effect of task similarity when interacting with (1) a human partner and (2) the computer partner. We found a significant effect of task similarity for individuals interacting with the computer partner, *F*(1,14) = 5.20, *p* = 0.04, *η*_*p*_^2^ = 0.27, but not with a human partner. Specifically, pianists performing with the computer partner exhibited a moderate synchronization (e.g., alpha power increase) in the “similar” task condition and a minor desynchronization (e.g., alpha power decrease) in the “dissimilar” task condition.

Figure 5B illustrates that the time course of alpha modulation in joining performers was different from that for starting performers because the joining performers finished playing measure four, rested for the first half of measure five, and then began to play again from the unison onset which occurred halfway through measure five. As a result, they often show a characteristic rebound around 0.5 s, followed by a desynchronization or return to baseline prior to beginning to play at the unison onset. A 2 × 2 × 3 × 8 ANOVA on alpha modulation in joining performers revealed no significant interactions between variables or main effects.

### Associations Between EQ, Note-Onset Asynchrony, and Alpha Modulation

We assessed possible associations between performer EQ, alpha modulation, and note-onset asynchrony behavior based on the aforementioned note-onset asynchrony and alpha modulation results. Consistent with the note-onset asynchrony and alpha modulation results presented, we analyzed data separately for individuals assuming starting vs. joining roles. Notably, we established that there was no significant difference in EQ between performers who arrived first (performing with the computer partner and then their human partner) and those who arrived second (performing with their human partner and then the computer partner).

#### Starting Role

Our analysis of note-onset asynchronies for performers in a starting role revealed a significant effect of performing with a human vs. computer partner on both average asynchronies and asynchrony variability. Namely, performers more frequently played notes ahead of the computer partner but exhibited greater variability of onset asynchronies with the human partner. Based on these results we first established the participant-wise human vs. computer condition difference (HvC difference), averaged across the similar vs. dissimilar task conditions, for both (1) average asynchronies and (2) asynchrony variability. We then compared each of these variables to (1) the HvC difference in alpha activity and (2) performer EQ. In comparing the note-onset asynchrony variables to the HvC difference in alpha activity we looked at associations with each of the individual electrode groups, as well as activity averaged across electrode groups. None of these correlations revealed significant associations between note-onset asynchrony behavior and alpha activity or performer EQ related to the difference in interaction with a human vs. computer partner.

Our analysis of alpha activity indicated a significant difference between the similar vs. dissimilar conditions for performers playing the starting role during interaction with the computer partner. We were interested in exploring this effect further by evaluating any potential associations between this difference and performer EQ as well as note-onset asynchrony behavior.

For each performer assuming the starting role and performing with the computer partner we first identified the difference in alpha activity between the similar vs. dissimilar conditions (SvD difference), collapsing across our three electrode groups. We also identified the SvD difference in the performer’s average asynchrony, as well as the SvD difference in the standard deviation of their asynchronies (i.e., the difference in asynchrony variability). We then used three separate correlations to evaluate the associations between the participant-wise SvD difference in alpha activity between conditions and each of (1) performer EQ, (2) the SvD difference in average asynchrony, and (3) the SvD difference in asynchrony variability. We did not observe significant associations between the participant-wise SvD difference in alpha activity and (1) performer EQ or (2) the SvD difference in average asynchrony. However, we did find a moderate, although non-significant, negative correlation between the SvD difference in alpha activity and the SvD difference in asynchrony variability, *r*(12) = −0.45, *p* = 0.11. This association indicates that the greater an individual’s alpha desynchronization in the “dissimilar” task condition as compared to the “similar” task condition, the greater the variability in their note-onset asynchronies for the “dissimilar” vs. the “similar” condition.

Given the possible association between SvD alpha activity and SvD asynchrony variability we also chose to evaluate the correlation between the SvD difference in asynchrony variability with the computer partner and performer EQ. This yielded a significant negative correlation, *r*(12) = −0.59, *p* = 0.03. This association indicates that individuals with a higher EQ showed greater variability in note-onset asynchronies in the “dissimilar” condition compared to the “similar” condition.

#### Joining Role

Our analysis of note-onset asynchrony behavior by joining performers revealed a significant difference in average asynchrony between playing with a human vs. computer partner, as well as an interaction between partner animacy and task similarity for asynchrony variability. Like we saw for starting performers, joining performers anticipated the computer partner more frequently than a human partner. They also exhibited greater asynchrony variability with human partners, with this difference being greater in the similar task condition compared to the dissimilar task condition. We calculated the participant-wise human vs. computer difference (HvC difference) in average asynchrony and compared this to (1) the HvC difference in alpha activity and (2) performer EQ. In examining the relationship between average asynchrony and alpha activity we looked at separate electrode groups, as well as activity averaged across electrode groups. None of these correlations revealed significant associations. We also calculated the participant-wise HvC difference in asynchrony variability and compared this variables to the (1) the HvC difference in alpha activity and (2) performer EQ. Neither of these associations were significant.

Our alpha activity analysis for joining performers did not reveal any significant interactions between variables or main effects so we did not examine further correlations with this data.

## Discussion

The current study is the first to systematically investigate how co-actor asymmetries act and interact to shape neural and behavioral activity during interpersonal interaction. Our findings indicate that asymmetries in musical role (starting vs. joining), task similarity (similar vs. dissimilar melodic parts), and performer animacy (human-to-human vs. human-to-non-adaptive computer) act to define specific interactive contexts within which performers experience characteristic relationships with their co-actor’s behavior. These characteristic relationships are reflected in the temporal dynamics of neural alpha modulation and behavioral coordination that each actor exhibits during a short period of musical unison.

Our observation of increased note-onset asynchrony during performance with a computer partner indicates a general discrepancy between human and computer timing dynamics. This is likely due to the lack of adaptability inherent to the computer partner in our study (Repp and Keller, 2008) and the natural human inclination to anticipate periodic stimuli (Mates et al., 1994). As noted above, we did not statistically compare neural or behavioral activity between the starting vs. joining role due to the difference in movement onset during the final unison phrase. However, a qualitative review of our findings as presented in Figure 3 suggests that in the computer condition both starters and joiners generally played ahead of the computer. Again, this likely relates to a human inclination to play faster than the set computer tempo.

In the human condition, joiners typically played just slightly ahead of starters on average. This result is similar to Goebl and Palmer’s (2009) observation that follower pianists tended to temporally precede leader pianists in conditions where they could hear the leader’s part of a duet as well as their own. This anticipation was small when the leader also heard the follower, and greater when the leader could only hear themselves. The roles in our study were most similar to the full auditory feedback condition in their study, with the exception that starters and joiners in our study alternated playing prior to the final unison phrase. As a result, the starter may have focused more on their own part, creating some similarity between our starter vs. joiner roles and the leader-follower condition in Goebl and Palmer’s study in which the leader only received auditory feedback corresponding to their own behavior. With this understanding of the relationship between co-performers, the observed temporal asynchrony is, in fact, consistent with the idea that joiners exhibited a small negative mean asynchrony (NMA) with respect to the starter after the starter played the first three notes of the phrase final unison phrase.

Interestingly, we observed reduced variability of note-onset asynchronies during interaction with the computer partner as compared to the human partner. These results are somewhat contrary to previous findings demonstrating that optimal levels of mutual adaptivity during synchronization lead to reduced variability (Fairhurst et al., 2012). We speculate that this discrepancy is driven by the fact that we evaluated coordination stability over only four key-presses, while the previous work looked at much longer sequences of synchronized taps. Given the dynamic nature of interpersonal interaction, measures of coordination averaged over a long time-window may be substantially different than coordinative patterns in a local segment.

For performers in the joining role we observed a consistent alpha synchronization followed by a desynchronization or return to baseline in alpha-band activity immediately prior to the unison. This finding could indicate that joining performers were preparing to start playing the unison, with alpha ERD reflecting movement preparation (Arroyo et al., 1993; Pfurtscheller et al., 1997; de Jong et al., 2006; Gladwin et al., 2008; Yamanaka and Yamamoto, 2010). At the same time, this may also reflect the joiner attending to their partner’s (i.e., starter’s) musical activity in order to achieve joint temporal coordination (Jin et al., 2006; Klimesch et al., 2007; Klimesch, 2012; Novembre et al., 2016). The time course of alpha modulation in joining performers was not significantly affected by having a similar vs. dissimilar musical task to one’s co-performer. Starting performers were already playing throughout the unison measure and generally displayed small alpha modulations around the unison onset. In some conditions they also displayed distinct alpha desynchronization prior to the unison onset, independent from alpha ERD that would have been associated with the start of their playing at the beginning of the measure.

As we saw for joining performers, for starting performers playing with a human partner there was no effect of musical task similarity on alpha modulation prior to unison. However, in the condition where the co-performer was the computer partner and their parts were musically similar we actually observed ERS, constituting significantly different alpha activity from the condition in which starters had a distinctly different musical part from the computer partner. While this result is somewhat consistent with previous work revealing that individuals exhibit greater alpha ERD during interaction with a human partner vs. a computer partner (Billeke et al., 2014), it also provides a more nuanced view of the effects of role asymmetries on neural processes related to perception and action. Specifically, musical task similarity appeared to moderate the effect of playing with a computer partner but only for performers assuming a starting role in the duet. We speculate that the experience of being a starting performer during interaction with a computer partner playing a similar part is akin to that of performing a solo with a karaoke accompaniment. Among all of the performance conditions created in the current study this situation may invoke the strongest solo mindset for a performer and therefore result in the least attentiveness to co-performer behavior. This view is supported by previous studies which have related ERS in the alpha band to the inhibition of external stimuli or co-actor activity during movement (Klimesch et al., 2007; Babiloni et al., 2012; Klimesch, 2012).

Notably, there was no effect of electrode grouping on starter or joiner alpha modulation, and no interaction between electrode grouping, performer animacy, and task similarity. The occurrence of consistent alpha modulation across left and right parietal and frontal-centro-medial areas is in line with the role of alpha as facilitating long-range communication for domain-general attentional functions. Such attention processes in turn would act to support the temporal coordination of social interactive behaviors in our task. This pattern is in contrast to the right centro-parietal alpha modulation specific to the subtle timing difference between performers in a joint tapping task (Tognoli et al., 2007). Also, the lack of lateralization in the centro-parietal sites here speaks against the relation to movement-related functions because our participants used only the right hand for keyboard playing.

In the current study we did not directly compare starter vs. joiner alpha activity because movement onsets differed between the two conditions. Because Konvalinka et al. (2014) found greater frontal alpha desynchronization in leaders compared to followers, one might expect a similar pattern of difference between our starting and joining conditions separate from the difference due to distinct movement onsets. However, it is important to note that our definition of starting and joining roles depended on the global music context rather than a local measure of adaptability of own movement interval to the partner’s previous interval, employed by Konvalinka et al. (2014), which fluctuated from time to time. Indeed, a visual comparison of our starter and joiner data did not reveal noticeable differences in the magnitude of alpha suppression. Together these findings indicate that the assignment to starter and joiner roles in our study did not have the same effect on co-actor attention as the emergent functional asymmetry at the beat-to-beat level between leaders and followers in the previous work. In other words, it is quite possible that the roles defined by musical turn-taking at the phrase-structure level in our design engage the brain in a different manner than asymmetrical roles identified at a local level. Also, the musical task in our study is considerably more challenging than synchronized finger-tapping and generally required greater attention to co-actor behavior for both starters and joiners.

The effects of partner animacy that we saw on behavioral patterns of note-onset asynchrony magnitude and variability were not associated with any systematic difference in alpha modulation. However, we did detect a moderate, non-significant correlation relating alpha modulation to note-onset asynchrony variability for the starting performer when interacting with the computer partner. Namely, the difference in alpha ERD between the similar and dissimilar musical task conditions was negatively associated with the difference in note-onset asynchrony between the two conditions. This association indicates that individuals who exhibited greater alpha suppression in the “dissimilar” task condition compared to the “similar” task condition also displayed greater variability of note-onset asynchronies in the “dissimilar” vs. “similar” condition. As we have noted this effect was only approaching significance, but it suggests that there may be some connection between attention to co-actor behavior and increased variability, possibly as the result of high levels of adaptation during interpersonal coordination when task similarity between co-actors plays a role.

Interestingly, we further identified a significant correlation between the difference in asynchrony variability in the “similar” and “dissimilar” conditions and performer EQ for starting performers interacting with a computer partner. This association established that individuals with a higher EQ exhibited greater asynchrony variability in the “dissimilar” condition compared to the “similar” condition. In both Pecenka and Keller’s (2011) work and our own study, increased perspective-taking or empathy therefore appears to be associated with a greater influence of external stimulus or co-actor activity on an individual’s temporal pattern of behavior. During tasks which involve temporal coordination, individuals respond to this influence by continuously acting to adapt their behavior to the ongoing stimulus or co-actor activity. As a result, their behavior becomes more variable than that of an individual who doesn’t exhibit the same degree of adaptivity. Thus, EQ is thought to correspond to the level of adaptivity an individual exhibits during interaction, with higher EQ correlating with greater adaptivity.

It is possible that we saw heightened variability specifically within the “dissimilar” condition because performers saw themselves as occupying a more distinct role than their co-performer, thus making their contribution to the ensemble somewhat more tangible. Our alpha ERD findings indicate that in the “similar,” computer condition performers were less attentive to the co-performer part, likely because they saw themselves as occupying a solo role and did not need to adapt or be adapted to in order to achieve a successful performance. However, once performers experience their parts as distinct and complementary, they may experience a greater need for adaptation between performers to achieve the joint goal. People with high levels of perspective-taking are more likely to respond to this context with an increase in adaptivity to their co-performer, while those with lower levels are more likely to maintain a more stable pattern of behavior.

Although we did not replicate the direct association between EQ and alpha ERD presented in previous work using a musical performance observation task (Babiloni et al., 2012), our results still suggest that there are strong connections between trait empathy, alpha modulation, and behavioral coordination during interpersonal interaction. Many existing studies have established an association between social interaction and alpha modulation, often suggesting that alpha modulation in MNS regions is involved in sensorimotor processing during social interactions (Babiloni et al., 2012; Sanger et al., 2013; Konvalinka et al., 2014; Novembre et al., 2016). However, to our knowledge, no existing work has illustrated how trait empathy might be related to alpha modulation itself. Our findings suggest that if a person has high trait empathy, perspective-taking may occur more naturally. In turn, this may be associated with more dynamic alpha modulation in response to the individual’s current task demands, and, depending on these conditions, more sensitivity in the action-perception of a co-actor’s behavior. Our study is also the first to capture the effects of multiple levels of co-performer asymmetries simultaneously in a controlled musical performance task. By always having participants perform an identical musical sequence in the unison phrase based on their starter or joiner role we are able to demonstrate that co-performer asymmetries in the priming context have a measurable impact on subsequent alpha modulation during the unison.

Future work can build on these findings to identify how specific levels of engagement, as indicated by alpha ERD, are dynamically linked to collective performance outcomes which are influenced by certain asymmetries between co-actors. This suggests that asymmetries between co-actors could also be adjusted systematically during real world interaction in order to achieve greater actor engagement. For example, based on our own findings, when people have to interact with computer-generated actors, the design of the computer co-actor should include features of adaptation to human co-actor’s behavior in order to support human engagement during interaction, especially if the two actors have similar task roles and the human initiates the task. Alternatively, when people are engaged in situations where certain asymmetries associated with lower engagement are unavoidable, other adjustments and strategies could be employed to increase engagement and improve performance. For instance, in the context of network ensemble music performance, long audio and video delays may prevent one performer from effectively adapting to their co-actor. The other individual might then deal with this issue by re-structuring the environment in order to remain engaged with their (apparently not so successfully adaptive) co-performer at key points during the interaction. In a musical context, such a solution might be as simple as adding a written reminder to attend to another group member’s behavior, or utilizing shared cues to overcome the timing discrepancies at a specific point in music. At a broader level, this could also involve training individuals to remain engaged with co-actor behavior, even when engagement is less likely to occur naturally, as enhancing an individual’s engagement is likely to improve collective performance.

Our study includes several noteworthy limitations and also illuminates areas for future work. First, our findings regarding the effect of partner animacy are consistent with previous work evaluating the effects of non-adaptivity during ensemble performance (e.g., Demos et al., 2017). However, we are somewhat limited in drawing conclusions about this effect given that in our human partner condition both co-performers were always able to see each other while in our computer partner condition there was no physically or visually presented co-actor. It is possible that the visual information about co-performer behavior available in the human partner condition afforded smaller average note-onset asynchronies and may have affected alpha band activity as well. Interestingly, however, there is also existing work showing that the absence of visual information during piano duet performance is associated with a higher degree of coupling between performers (Walton et al., 2015). In this case the authors suggest that musicians become more tightly coordinated in order to increase the likelihood of cohesive performance. Future work should aim to establish a deeper understanding of the independent effects of co-performer non-adaptivity and visual information about co-performer behavior. It is worth noting, however, that our key results were observed within each partner condition. This means that even when performers experienced the same visual environment they experienced the duet performance differently depending on the musical similarity between the two parts.

Second, while we demonstrated a relationship between EQ and note-onset asynchrony variability within individual performers, our study was not designed to evaluate the effect of asymmetries between co-performers’ persistent, social personality traits or behavioral characteristics. Extensions of our present work could be used to identify associations between pairwise asymmetries in characteristics like empathy and locus of control and (1) co-actor differences in behavioral and neural activity or (2) collective coordination outcomes. Recent work has revealed that individuals with expertise in couple dancing (e.g., Tango, Salsa, Swing) show enhanced neural activity when they perform the role for which they are an expert (i.e., leader vs. follower) (Chauvigné and Brown, 2018). These findings indicate that trait-level asymmetries between co-actors significantly affect each actor’s neural and behavioral processes during interpersonal interaction.

Our role asymmetries existed at the musical phrase level context, which is notably shorter-term than the trait level context. However, it is important to remember that asymmetries existing at even shorter timescales are also likely to influence the temporal dynamics of interpersonal coordination. In many social interactions, musical and otherwise, co-actor asymmetries are dynamically varied. As a result, shifts in attention and related patterns of coordination are likely to fluctuate frequently. This occurs in other creative social interactions like dance and acting, especially those which allow for some degree of improvisation, as well team or unit-based scenarios like sporting events and military missions.

The continued, simultaneous use of neural and behavioral measurement techniques will allow future researchers to further investigate the ability of individuals to adapt to changing asymmetries while maintaining coordinated activity. Notably, our focus in this study was on the neural and behavioral activity of two co-performers during interaction and did not include consideration of a larger group of co-actors or the experience of a listener or observer. It would, for example, be valuable to determine whether the kind of causal influences on respiration and heart rate variability Müller and Lindenberger (2011) observed between a conductor and choir members are also related to distinct conductor vs. choir member alpha modulation. Additionally, future work exploring the association between co-performer alpha modulation and listener or observer experience will provide critical insight into how co-actor engagement is facilitated by rapid modulations of neural activity and may shape a third-party audience’s perception of collaborative performance.

## Data Availability Statement

The datasets generated for this study are available on request to the corresponding author.

## Ethics Statement

This study was approved by and carried out in accordance with the recommendations of the Stanford University Institutional Review Board. All participants gave written informed consent.

## Author Contributions

All authors designed the study and edited the manuscript. MW created the experimental apparatus and MH and AW verified its setup. TF, IR, MH, NG, TD, WR, and CN collected the data. AW, IR, MH, and TF analyzed the data. AW and TF wrote the manuscript.

## Conflict of Interest

The authors declare that the research was conducted in the absence of any commercial or financial relationships that could be construed as a potential conflict of interest.

## References

[B1] Ahn S. Cho H. Kwon M. Kim K. Kwon H. Kim B. S. (2018). Interbrain phase synchronization during turn-taking verbal interaction—a hyperscanning study using simultaneous EEG/MEG. *Hum. Brain Mapp.* 39 171–188. 10.1002/hbm.23834 29024193 PMC6866597

[B2] Arnstein D. Cui F. Keysers C. Maurits N. M. Gazzola V. (2011). μ-suppression during action observation and execution correlates with BOLD in dorsal premotor, inferior parietal, and SI cortices. *J. Neurosci.* 31 14243–14249. 10.1523/jneurosci.0963-11.2011 21976509 PMC6623646

[B3] Arroyo S. Lesser R. P. Gordon B. Uematsu S. Jackson D. Webber R. (1993). Functional significance of the mu rhythm of human cortex: an electrophysiologic study with subdural electrodes. *Electroencephalogr. Clin. Neurophysiol.* 87 76–87. 10.1016/0013-4694(93)90114-b 7691544

[B4] Babiloni C. Buffo P. Vecchio F. Marzano N. Del Percio C. Spada D. (2012). Brains “in concert”: frontal oscillatory alpha rhythms and empathy in professional musicians. *Neuroimage* 60 105–116. 10.1016/j.neuroimage.2011.12.008 22186679

[B5] Babiloni C. Carducci F. Cincotti F. Rossini P. M. Neuper C. Pfurtscheller G. (1999). Human movement-related potentials vs desynchronization of EEG alpha rhythm: a high-resolution EEG study. *Neuroimage* 10 658–665. 10.1006/nimg.1999.0504 10600411

[B6] Babiloni C. Del Percio C. Rossini P. M. Marzano N. Iacoboni M. Infarinato F. (2009). Judgment of actions in experts: a high-resolution EEG study in elite athletes. *Neuroimage* 45 512–521. 10.1016/j.neuroimage.2008.11.035 19111623

[B7] Babiloni C. Marzano N. Infarinato F. Iacoboni M. Rizza G. Aschieri P. (2010). “Neural efficiency” of experts’ brain during judgment of actions: a high-resolution EEG study in elite and amateur karate athletes. *Behav. Brain Res.* 207 466–475. 10.1016/j.bbr.2009.10.034 19891991

[B8] Baron-Cohen S. Wheelwright S. (2004). The empathy quotient: an investigation of adults with Asperger syndrome or high functioning autism, and normal sex differences. *J. Autism Dev. Disord.* 34 163–175. 10.1023/b:jadd.0000022607.19833.00 15162935

[B9] Billeke P. Zamorano F. Chavez M. Cosmelli D. Aboitiz F. (2014). Functional cortical network in alpha band correlates with social bargaining. *PLoS One* 9:e109829. 10.1371/journal.pone.0109829 25286240 PMC4186879

[B10] Byblow W. D. Chua R. Goodman D. (1995). Asymmetries in coupling dynamics of perception and action. *J. Motor Behav.* 27 123–137. 10.1080/00222895.1995.9941705 12736122

[B11] Cannon E. N. Yoo K. H. Vanderwert R. E. Ferrari P. F. Woodward A. L. Fox N. A. (2014). Action experience, more than observation, influences mu rhythm desynchronization. *PLoS One* 9:e92002. 10.1371/journal.pone.0092002 24663967 PMC3963876

[B12] Chang A. Livingstone S. R. Bosnyak D. J. Trainor L. J. (2017). Body sway reflects leadership in joint music performance. *Proc. Natl. Acad. Sci. U.S.A.* 114 E4134–E4141. 10.1073/pnas.1617657114 28484007 PMC5448222

[B13] Chauvigné L. A. Brown S. (2018). Role-specific brain activations in leaders and followers during joint action. *Front. Hum. Neurosci.* 12:401. 10.3389/fnhum.2018.00401 30349467 PMC6186800

[B14] Cochin S. Barthelemy C. Lejeune B. Roux S. Martineau J. (1998). Perception of motion and qEEG activity in human adults. *Electroencephalogr. Clin. Neurophysiol.* 107 287–295. 10.1016/s0013-4694(98)00071-6 9872446

[B15] Cochin S. Barthelemy C. Roux S. Martineau J. (2001). Electroencephalographic activity during perception of motion in childhood. *Eur. J. Neurosci.* 13 1791–1796. 10.1046/j.0953-816x.2001.01544.x 11359530

[B16] Cui X. Bryant D. M. Reiss A. L. (2012). NIRS-based hyperscanning reveals increased interpersonal coherence in superior frontal cortex during cooperation. *Neuroimage* 59 2430–2437. 10.1016/j.neuroimage.2011.09.003 21933717 PMC3254802

[B17] de Jong R. Gladwin T. E.’t Hart B. M. (2006). Movement-related EEG indices of preparation in task switching and motor control. *Brain Res.* 1105 73–82. 10.1016/j.brainres.2006.03.030 16630582

[B18] Demos A. P. Carter D. J. Wanderley M. M. Palmer C. (2017). The unresponsive partner: roles of social status, auditory feedback, and animacy in coordination of joint music performance. *Front. Psychol.* 8:149. 10.3389/fpsyg.2017.00149 28261123 PMC5306131

[B19] Dumas G. Martinerie J. Soussignan R. Nadel J. (2012). Does the brain know who is at the origin of what in an imitative interaction? *Front. Hum. Neurosci.* 6:128. 10.3389/fnhum.2012.00128 22582043 PMC3348720

[B20] Dumas G. Nadel J. Soussignan R. Martinerie J. Garnero L. (2010). Inter-brain synchronization during social interaction. *PLoS One* 5:e12166. 10.1371/journal.pone.0012166 20808907 PMC2923151

[B21] Engel A. Keller P. E. (2011). The perception of musical spontaneity in improvised and imitated jazz performances. *Front. Psychol.* 2:83. 10.3389/fpsyg.2011.00083 21738518 PMC3125527

[B22] Fairhurst M. T. Janata P. Keller P. (2012). Being and feeling in sync with an adaptive virtual partner: brain mechanisms underlying dynamic cooperativity. *Cereb. Cortex* 23 2592–2600. 10.1093/cercor/bhs243 22892422

[B23] Fairhurst M. T. Janata P. Keller P. E. (2013). Being and feeling in sync with an adaptive virtual partner: brain mechanisms underlying dynamic cooperativity. *Cereb. Cortex* 23 2592–2600. 10.1093/cercor/bhs243 22892422

[B24] Frenkel-Toledo S. Bentin S. Perry A. Liebermann D. G. Soroker N. (2014). Mirror-neuron system recruitment by action observation: effects of focal brain damage on mu suppression. *NeuroImage* 87 127–137. 10.1016/j.neuroimage.2013.10.019 24140938

[B25] Gastaut H. J. Bert J. (1954). EEG changes during cinematographic presentation (Moving picture activation of the EEG). *Electroencephalogr. Clin. Neurophysiol.* 6 433–444. 10.1016/0013-4694(54)90058-9 13200415

[B26] Giese M. A. Dijkstra T. M. H. Schöner G. Gielen C. C. A. M. (1996). Identification of the nonlinear state-space dynamics of the action-perception cycle for visually induced postural sway. *Biol. Cybernet.* 74 427–437. 10.1007/s004220050254 8991458

[B27] Gladwin T. E.’t Hart B. M. de Jong R. (2008). Dissociations between motor-related EEG measures in a cued movement sequence task. *Cortex* 44 521–536. 10.1016/j.cortex.2007.10.005 18387585

[B28] Goebl W. Palmer C. (2009). Synchronization of timing and motion among performing musicians. *Music Percept. Interdiscipl. J.* 26 427–438. 10.1525/mp.2009.26.5.427

[B29] Hari R. Forss N. Avikainen S. Kirveskari E. Salenius S. Rizzolatti G. (1998). Activation of human primary motor cortex during action observation: a neuromagnetic study. *Proc. Natl. Acad. Sci. U.S.A.* 95 15061–15065. 10.1073/pnas.95.25.15061 9844015 PMC24575

[B30] Hari R. Kujala M. V. (2009). Brain basis of human social interaction: from concepts to brain imaging. *Physiol. Rev.* 89 453–479. 10.1152/physrev.00041.2007 19342612

[B31] Hobson H. M. Bishop D. V. M. (2016). Mu suppression–a good measure of the human mirror neuron system? *Cortex* 82 290–310. 10.1016/j.cortex.2016.03.019 27180217 PMC4981432

[B32] Holz E. M. Doppelmayr M. Klimesch W. Sauseng P. (2008). EEG correlates of action observation in humans. *Brain Topogr.* 21 93–99. 10.1007/s10548-008-0066-1 18780176

[B33] Huberth M. Dauer T. Nanou C. Román I. Gang N. Reid W. (2019). Performance monitoring of self and other in a turn-taking piano duet: a dual-EEG study. *Soc. Neurosci.* 14 449–461. 10.1080/17470919.2018.1492968 29938589

[B34] Iacoboni M. Dapretto M. (2006). The mirror neuron system and the consequences of its dysfunction. *Nat. Rev. Neurosci.* 7 942–951. 10.1038/nrn2024 17115076

[B35] Jeka J. Oie K. Schöner G. Dijkstra T. Henson E. (1998). Position and velocity coupling of postural sway to somatosensory drive. *J. Neurophysiol.* 79 1661–1674. 10.1152/jn.1998.79.4.1661 9535937

[B36] Jin Y. O’Halloran J. P. Plon L. Sandman C. A. Potkin S. G. (2006). Alpha EEG predicts visual reaction time. *Int. J. Neurosci.* 116 1035–1044. 10.1080/00207450600553232 16861166

[B37] Jurkiewicz M. T. Gaetz W. C. Bostan A. C. Cheyne D. (2006). Post-movement beta rebound is generated in motor cortex: evidence from neuromagnetic recordings. *Neuroimage* 32 1281–1289. 10.1016/j.neuroimage.2006.06.005 16863693

[B38] Keil A. Debener S. Gratton G. Junghöfer M. Kappenman E. S. Luck S. J. (2014). Committee report: publication guidelines and recommendations for studies using electroencephalography and magnetoencephalography. *Psychophysiology* 51 1–21. 10.1111/psyp.12147 24147581

[B39] Keller P. E. (2008). “Joint action in music performance,” in *Enacting Intersubjectivity: A Cognitive and Social Perspective to the Study of Interactions*, eds Morganti F. Carassa A. Riva G. (Amsterdam: IOS Press), 205–221.

[B40] Kelso J. A. S. DelColle J. D. Schöner G. (1990). Action-perception as a pattern formation process. *Attent. Perform.* 5 139–169. 10.1007/s11571-006-9001-x 19003492 PMC2288954

[B41] Klimesch W. (2012). Alpha-band oscillations, attention, and controlled access to stored information. *Trends Cogn. Sci.* 16 606–617. 10.1016/j.tics.2012.10.007 23141428 PMC3507158

[B42] Klimesch W. Doppelmayr M. Russegger H. Pachinger T. Schwaiger J. (1998). Induced alpha band power changes in the human EEG and attention. *Neurosci. Lett.* 244 73–76. 10.1016/s0304-3940(98)00122-0 9572588

[B43] Klimesch W. Sauseng P. Hanslmayr S. (2007). EEG alpha oscillations: the inhibition–timing hypothesis. *Brain Res. Rev.* 53 63–88. 10.1016/j.brainresrev.2006.06.003 16887192

[B44] Konvalinka I. Bauer M. Stahlhut C. Hansen L. K. Roepstorff A. Frith C. D. (2014). Frontal alpha oscillations distinguish leaders from followers: multivariate decoding of mutually interacting brains. *Neuroimage* 94 79–88. 10.1016/j.neuroimage.2014.03.003 24631790

[B45] Lachat F. Hugueville L. Lemaréchal J.-D. Conty L. George N. (2012). Oscillatory brain correlates of live joint attention: a dual-EEG study. *Front. Hum. Neurosci.* 6:156. 10.3389/fnhum.2012.00156 22675297 PMC3365444

[B46] Loehr J. D. Palmer C. (2011). Temporal coordination between performing musicians. *Q. J. Exp. Psychol.* 64 2153–2167. 10.1080/17470218.2011.603427 21929475

[B47] Martineau J. Cochin S. (2003). Visual perception in children: human, animal and virtual movement activates different cortical areas. *Int. J. Psychophysiol.* 51 37–44. 10.1016/s0167-8760(03)00151-x 14629921

[B48] Mates J. Müller U. Radil T. Pöppel E. (1994). Temporal integration in sensorimotor synchronization. *J. Cogn. Neurosci.* 6 332–340. 10.1162/jocn.1994.6.4.332 23961729

[B49] Ménoret M. Varnet L. Fargier R. Cheylus A. Curie A. Des Portes V. (2014). Neural correlates of non-verbal social interactions: a dual-EEG study. *Neuropsychologia* 55 85–97. 10.1016/j.neuropsychologia.2013.10.001 24157538

[B50] Müller V. Lindenberger U. (2011). Cardiac and respiratory patterns synchronize between persons during choir singing. *PLoS One* 6:e24893. 10.1371/journal.pone.0024893 21957466 PMC3177845

[B51] Naeem M. Prasad G. Watson D. R. Kelso J. S. (2012a). Electrophysiological signatures of intentional social coordination in the 10–12 Hz range. *Neuroimage* 59 1795–1803. 10.1016/j.neuroimage.2011.08.010 21871572

[B52] Naeem M. Prasad G. Watson D. R. Kelso J. S. (2012b). Functional dissociation of brain rhythms in social coordination. *Clin. Neurophysiol.* 123 1789–1797. 10.1016/j.clinph.2012.02.065 22425484

[B53] Novembre G. Sammler D. Keller P. (2016). Neural alpha oscillations index the balance between self-other integration and segregation in real-time joint action. *Neuropsychologia* 89 414–425. 10.1016/j.neuropsychologia.2016.07.027 27449708

[B54] Oberman L. M. Pineda J. A. Ramachandran V. S. (2007). The human mirror neuron system: a link between action observation and social skills. *Soc. Cogn. Affect. Neurosci.* 2 62–66. 10.1093/scan/nsl022 18985120 PMC2555434

[B55] Orgs G. Dombrowski J. H. Heil M. Jansen-Osmann P. (2008). Expertise in dance modulates alpha/beta event-related desynchronization during action observation. *Eur. J. Neurosci.* 27 3380–3384. 10.1111/j.1460-9568.2008.06271.x 18598273

[B56] Pecenka N. Keller P. E. (2011). The role of temporal prediction abilities in interpersonal sensorimotor synchronization. *Exp. Brain Res.* 211 505–515. 10.1007/s00221-011-2616-0 21424257

[B57] Perry A. Bentin S. (2009). Mirror activity in the human brain while observing hand movements: a comparison between EEG desynchronization in the mu-range and previous fMRI results. *Brain Res.* 1282 126–132. 10.1016/j.brainres.2009.05.059 19500557

[B58] Pfurtscheller G. (1992). Event-related synchronization (ERS): an electrophysiological correlate of cortical areas at rest. *Electroencephalogr. Clin. Neurophysiol.* 83 62–69. 10.1016/0013-4694(92)90133-3 1376667

[B59] Pfurtscheller G. (2003). Induced oscillations in the alpha band: functional meaning. *Epilepsia* 44 2–8. 10.1111/j.0013-9580.2003.12001.x 14641556

[B60] Pfurtscheller G. Brunner C. Schlögl A. Da Silva F. L. (2006). Mu rhythm (de) synchronization and EEG single-trial classification of different motor imagery tasks. *NeuroImage* 31 153–159. 10.1016/j.neuroimage.2005.12.003 16443377

[B61] Pfurtscheller G. Neuper C. Andrew C. Edlinger G. (1997). Foot and hand area mu rhythms. *Int. J. Psychophysiol.* 26 121–135. 10.1016/s0167-8760(97)00760-5 9202999

[B62] Quandt L. C. Marshall P. J. (2014). The effect of action experience on sensorimotor EEG rhythms during action observation. *Neuropsychologia* 56 401–408. 10.1016/j.neuropsychologia.2014.02.015 24568874 PMC4009369

[B63] Repp B. H. (2006). Does an auditory distractor sequence affect self-paced tapping? *Acta Psychol.* 12 81–107. 10.1016/j.actpsy.2005.06.006 16098944

[B64] Repp B. H. Keller P. (2008). Sensorimotor synchronization with adaptively timed sequences. *Hum. Move. Sci.* 27 423–456. 10.1016/j.humov.2008.02.016 18405989

[B65] Repp B. H. Penel A. (2004). Rhythmic movement is attracted more strongly to auditory than to visual rhythms. *Psychol. Res.* 68 252–270. 12955504 10.1007/s00426-003-0143-8

[B66] Richardson M. J. Marsh K. L. Schmidt R. C. (2005). Effects of visual and verbal interaction on unintentional interpersonal coordination. *J. Exp. Psychol. Hum. Percept. Perform.* 31 62–79.4. 15709863 10.1037/0096-1523.31.1.62

[B67] Richardson M. J. Washburn A. Kallen R. W. Harrison S. J. (2016). “Symmetry and the behavioral dynamics of social coordination,” in *Interpersonal Coordination and Performance in Social Systems*, eds Passos P. Davis K. (Abingdon: Routledge), 65–81.

[B68] Russell D. M. Sternad D. (2001). Sinusoidal visuomotor tracking: intermittent servo-control or coupled oscillations? *J. Motor Behav.* 33 329–349. 10.1080/00222890109601918 11734409

[B69] Salmelin R. Hämäläinen M. Kajola M. Hari R. (1995). Functional segregation of movement-related rhythmic activity in the human brain. *Neuroimage* 2 237–243. 10.1006/nimg.1995.1031 9343608

[B70] Sanger J. Muller V. Lindenberger U. (2013). Directionality in hyperbrain networks discriminates between leaders and followers in guitar duets. *Front. Hum. Neurosci.* 7:234. 10.3389/fnhum.2013.00234 23761745 PMC3671173

[B71] Schmidt R. C. O’Brien B. (1997). Evaluating the dynamics of unintended interpersonal coordination. *Ecol. Psychol.* 9 189–206. 10.1207/s15326969eco0903_2

[B72] Schmidt R. C. Richardson M. J. Arsenault C. Galantucci B. (2007). Visual tracking and entrainment to an environmental rhythm. *J. Exp. Psychol. Hum. Percept. Perform.* 33 860–870. 10.1037/0096-1523.33.4.860 17683233

[B73] Schmidt R. C. Turvey M. T. (1994). Phase-entrainment dynamics of visually coupled rhythmic movements. *Biol. Cybernet.* 70 369–376. 10.1007/s004220050040 8148414

[B74] Skewes J. C. Skewes L. Michael J. Konvalinka I. (2015). Synchronised and complementary coordination mechanisms in an asymmetric joint aiming task. *Exp. Brain Res.* 233 551–565. 10.1007/s00221-014-4135-2 25362518

[B75] Tadel F. Baillet S. Mosher J. C. Pantazis D. Leahy R. M. (2011). Brainstorm: a user-friendly application for MEG/EEG analysis. *Comput. Intell. Neurosci.* 2011 1–13. 10.1155/2011/879716 21584256 PMC3090754

[B76] Taniguchi M. Kato A. Fujita N. Hirata M. Tanaka H. Kihara T. (2000). Movement-related desynchronization of the cerebral cortex studied with spatially filtered magnetoencephalography. *Neuroimage* 12 298–306. 10.1006/nimg.2000.0611 10944412

[B77] Tognoli E. Lagarde J. DeGuzman G. C. Kelso J. S. (2007). The phi complex as a neuromarker of human social coordination. *Proc. Natl. Acad. Sci. U.S.A.* 104 8190–8195. 10.1073/pnas.0611453104 17470821 PMC1859993

[B78] Walton A. Richardson M. J. Langland-Hassan P. Chemero A. Washburn A. (2015). “Musical improvisation: multi-scaled spatiotemporal patterns of coordination,” in *Proceedings of the 37th Annual Meeting of the Cognitive Science Society*, eds Noelle D. C. Dale R. Warlaumont A. S. Yoshimi J. Matlock T. Jennings C. D. (Austin, TX: Cognitive Science Society), 2595–2600.

[B79] Washburn A. Coey C. A. Romero V. Richardson M. J. (2014). Visual multifrequency entrainment: can 1: 2, 2: 3, and 3: 4 coordination occur spontaneously? *J. Motor Behav.* 46 247–257. 10.1080/00222895.2014.893980 24731065

[B80] Washburn A. Kallen R. W. Coey C. A. Shockley K. Richardson M. J. (2015). Harmony from chaos? Perceptual-motor delays enhance behavioral anticipation in social interaction. *J. Exp. Psychol. Hum. Percept. Perform.* 41 1166–1177. 10.1037/xhp0000080 26030437 PMC4516696

[B81] Wilson A. D. Collins D. R. Bingham G. P. (2005). Perceptual coupling in rhythmic movement coordination: stable perception leads to stable action. *Exp. Brain Res.* 164 517–528. 10.1007/s00221-005-2272-3 15887008

[B82] Wimmers R. H. Beek P. J. van Wieringen P. C. (1992). Phase transitions in rhythmic tracking movements: a case of unilateral coupling. *Hum. Move. Sci.* 11 217–226. 10.1016/0167-9457(92)90062-g

[B83] Wing A. M. Endo S. Bradbury A. Vorberg D. (2014). Optimal feedback correction in string quartet synchronization. *J. R. Soc. Interface* 93 20131125. 10.1098/rsif.2013.1125 24478285 PMC3928944

[B84] Wright M. Cassidy R. J. Zbyszyński M. F. (2004). “Audio and gesture latency measurements on Linux and OSX,” in *Proceedings of the International Computer Music Conference*, (Miami, FL: International Computer Music Association), 423–429.

[B85] Yamanaka K. Yamamoto Y. (2010). Lateralised EEG power and phase dynamics related to motor response execution. *Clin. Neurophysiol.* 121 1711–1718. 10.1016/j.clinph.2010.03.027 20434947

[B86] Zamm A. Pfordresher P. Q. Palmer C. (2015). Temporal coordination in joint music performance: effects of endogenous rhythms and auditory feedback. *Exp. Brain Res.* 233 607–615. 10.1007/s00221-014-4140-5 25399244 PMC4295031

